# CRISPRi: a way to integrate iPSC-derived neuronal models

**DOI:** 10.1042/BST20230190

**Published:** 2024-03-25

**Authors:** Sarah N.J. Franks, Rachel Heon-Roberts, Brent J. Ryan

**Affiliations:** 1 Oxford Parkinson's Disease Centre and Department of Physiology, Anatomy and Genetics, University of Oxford, Oxford OX1 3QU, UK; 2 Kavli Institute for Nanoscience Discovery, Dorothy Crowfoot Hodgkin Building, University of Oxford, Oxford OX1 3QU, UK

**Keywords:** CRISPR, functional genomics, high-throughput screening, induced pluripotent stem cells, neurodegeneration, Parkinson's disease

## Abstract

The genetic landscape of neurodegenerative diseases encompasses genes affecting multiple cellular pathways which exert effects in an array of neuronal and glial cell-types. Deconvolution of the roles of genes implicated in disease and the effects of disease-associated variants remains a vital step in the understanding of neurodegeneration and the development of therapeutics. Disease modelling using patient induced pluripotent stem cells (iPSCs) has enabled the generation of key cell-types associated with disease whilst maintaining the genomic variants that predispose to neurodegeneration. The use of CRISPR interference (CRISPRi), alongside other CRISPR-perturbations, allows the modelling of the effects of these disease-associated variants or identifying genes which modify disease phenotypes. This review summarises the current applications of CRISPRi in iPSC-derived neuronal models, such as fluorescence-activated cell sorting (FACS)-based screens, and discusses the future opportunities for disease modelling, identification of disease risk modifiers and target/drug discovery in neurodegeneration.

## Introduction

The advent of clustered, regularly interspersed, short palindromic repeat (CRISPR) technologies has rapidly changed the accessibility of genome editing, providing the ability to knockout genes or induce/correct point mutations. More recent catalytically inactive versions of the Cas9 protein provide the ability to modulate gene expression with genomic and temporal specificity. In this review, we provide an overview of how gene knockdown with CRISPR interference (CRISPRi) can (and will) be used as a powerful and complementary tool to integrate induced pluripotent stem cell (iPSC)-derived patient neuronal models, in order to understand the genetic drivers of neurodegeneration and find new drug targets and therapies.

CRISPRs are short, repetitive DNA sequences that form the basis of the bacterial immune system. In conjunction with CRISPR-associated (Cas) proteins, these sequences are transcribed and processed to form a ribonucleoprotein (RNP) complex that recognises and introduces double-stranded breaks into foreign DNA (reviewed in [1]). The potential for the use of CRISPR/Cas9 for genomic engineering started to emerge when multiple studies independently demonstrated the practicality and power of the system to engineer genomes [2–4]. These observations were quickly followed by several genome-wide genetic screening studies in several mammalian cell types [5–8]. Despite the success of the nuclease-active CRISPR/Cas9 system (henceforth, CRISPRn), it was discovered that targeting endogenous loci in bacteria and human cells produced a negative selection pressure [9–12]. Inactivating the nuclease activity of Cas9 (dCas9) maintained a level of gene silencing [9], without effect on proliferation [12].

## Development of CRISPRi

Initial iterations of CRISPRi technology relied on steric hindrance to block transcription initiation and elongation and showed low levels of gene knockdown in human cells. Gilbert et al. [13] fused the transcriptional repressor domain Krüppel associated box (KRAB) of the KOX1 transcription factor to the dCas9 protein, which resulted in increased silencing of gene expression that was maintained over time in culture. Further improvements were made to the system by refinements in the CRISPR/dCas9 system [14], including optimising the potency of single guide RNAs (sgRNAs), allowing for highly effective genome-wide libraries to be constructed [15–17], maintaining dCas9 expression over time [15] and the addition of repressive domains (e.g. SIN3A interacting domain (SID)) [18]. Since the development of CRISPRi, targeted and genome-wide CRISPRi screens have been conducted in both immortalised cell lines and human iPSC-derived neurons [19–22].

## CRISPR derivatives

In recent years, the dCas9 and Cas9 proteins have been engineered to attach several other transcription regulation domains. For example, fusion of four copies of the transcriptional activator VP16 led to CRISPR activation (CRISPRa), where genes targeted by sgRNAs are up-regulated [13]. Attaching epigenomic histone methylation and demethylation domains produced CRISPRoff and CRISPRon, respectively, allowing for the heritable silencing or activation of loci [23,24]. These technologies alone or paired with high-content readouts such as transcriptomics or proteomics allow for the interrogation of a broad range of biologically relevant questions.

## Why CRISPRi?

CRISPRi has multiple advantages over the pre-dating RNA interference (RNAi) technology. It is highly specific, with fewer off-target effects [9,25–28]. CRISPRi screens are also more reproducible [6,7], as well as being overall more effective [29]. Due to its action in repressing gene transcription in the nucleus, it can also be targeted to promoters, enhancers and other genomic regulatory regions, which enables a wider range of genetic elements to be screened.

Unlike RNAi, however, CRISPRi is most effective when using cell lines genetically engineered to express dCas9, or lines that can be easily transduced with lentiviral vectors expressing the small sgRNA and much larger dCas9. RNAi technology relies on endogenous systems to effectuate gene silencing, allowing for the simple delivery of small hairpin RNAs (shRNAs) into more varied cell types, such as patient-derived iPSCs. Importantly, the engineering of dCas9 and/or the sgRNA cassette provides the opportunity to express antibiotic resistance or fluorescent markers which can provide downstream utility.

Depending on the application, CRISPRi may also be advantageous over CRISPRn. CRISPRn produces different mutations in each cell, resulting in a mixed population of cells with various levels of gene expression, gene splicing and/or protein activity. CRISPRi does not induce permanent genome modification, allowing for reversible gene silencing [30], and due to the lack of nuclease activity, does not result in the accumulation of off-target mutations. The non-permanent nature of CRISPRi silencing is advantageous for longitudinal expression, as CRISPRi silencing shows less compensation than CRISPRn [31]. Moreover, CRISPRi is better suited to the functional study of essential genes which may induce cell death when mutated by CRISPRn [8].

However, there are limitations to be considered before the implementation of CRISPRi. First, the efficacy of gene silencing is highly dependent on targeting the dCas9–sgRNA complex to a narrow window (−50 to 300 bp) around the transcription start site (TSS) [15]. Thus, genes that possess multiple TSSs or cryptic TSSs cannot be targeted using a single sgRNA and may show poor repression of gene expression by CRISPRi. Second, it has been shown that CRISPRi is not strand specific [32] and targeting loci bearing a bidirectional promoter, can result in off-target silencing. Finally, by inhibiting transcription initiation, CRISPRi is effective at silencing isoforms that arise from alternative splicing but offers little flexibility in modulating the levels of such isoforms. In this context, CRISPRn can be advantageous in its ability to precisely modify splice sites or allow the removal of exons [33], enabling the up-regulation or down-regulation of specific isoforms.

The transient nature of CRISPRi in comparison with the permanence of CRISPRn is in some cases an advantage, but also presents some technical challenges. To maintain the silencing of genes, the expression of both dCas9 and sgRNA must be maintained. This can prove difficult over the course of neuronal differentiation, which can result in silencing of the CRISPRi machinery over extended culturing time. Alternatively, with CRISPRn, RNPs may be used transiently but result in long-lasting editing. Epigenomic silencing (CRISPRoff) has made it possible to permanently silence loci, thus retaining the advantages of gene silencing as opposed to gene modification [23]. It should also be noted that gene repression efficacy in CRISPRi is dependent on dCas9 expression [34] and cell-to-cell variability of machinery expression, such as in the case of a polyclonal dCas9-expressing cell line [35], may result in partial and variable gene silencing, which in turn increases variability in phenotypic readouts. Finally, unlike CRISPRa, CRISPRi provides no information on genes that are not endogenously expressed in the cell line or cell type of interest.

## Generation of iPSC-derived neurons

The ground-breaking discovery by Takahashi and Yamanaka in 2006 demonstrated the reprogramming of a mature somatic cell into an iPSC, first in mice [36], then in humans [37]. Through the ectopic expression of four transcription factors (Oct3/4, Sox2, Klf4, c-Myc), since dubbed ‘Yamanaka factors’, fibroblasts were reprogrammed into iPSCs, exhibiting comparable morphology, proliferation and pluripotency to an embryonic stem cell. The advent of iPSC technology has enabled the differentiation of various neuronal subtypes including dopaminergic [38], glutamatergic [39] and cholinergic neurons [40], in addition to important tissue-specific glial cell types including astrocytes [41] and microglia [42–46].

Significantly, iPSC-derived neurons from patients maintain their genetic background and disease-causing mutations, making them important models for understanding genetic contributions to disease in specific cellular subtypes of interest such as in Parkinson's disease (PD) [42–47], Alzheimer's disease (AD) [48], Huntington's disease (HD) [49], spinal muscular atrophy [50], amyotrophic lateral sclerosis (ALS) [40,51] and neurological disorders such as autism spectrum disorder [52], Rett syndrome [53] and schizophrenia [54]. Importantly, iPSC reprogramming may reset crucial ageing signatures such as epigenetic memory and metabolic ageing, implicating the translatability of iPSC-derived neurons to age-related neurodegenerative disease, compared with directly induced neurons (reviewed in [55,56]).

## Disease models: primary cultures and immortalised cell lines

iPSC-derived neurons offer several advantages over other *in vitro* human disease models. Human primary neuronal cultures are hindered by their infrequent accessibility and limited post-differentiation expandability. Contrastingly, many published CRISPR-based screens utilise immortalised cell lines, which provide an indefinite source of cells and whose culture is cost, time and resource-efficient [57]. However, although of human origin, immortalised cell lines frequently share greater genomic, metabolic, morphological and transcriptional biological similarities to the cancer cells from which they were derived, than to mature neurons [58]. This is exemplified by Sanchez et al. [59] who found that from the top 43 genes that modulate endogenous tau levels in a genome-wide CRISPRn screen in SH-SY5Y, five validated in iPSC-derived neurons, and only *TSC1* finally validated *in vivo*, demonstrating the benefit of cell lines as a springboard to complement iPSC and animal models of disease.

## Functional advantages of iPSC-derived neurons

Of the models described, iPSC-derived neurons have the most physiologically relevant biology for recapitulating human neuron physiology as seen *in vivo*. Once differentiated, iPSC-derived neurons are post-mitotic, metabolically switch to favour oxidative phosphorylation over glycolysis [60] and the transcriptome and proteome are extensively remodelled to reflect its neuronal identity [61]. Finally, some iPSC-derived neurons demonstrate functional hallmarks of mature neurons [62,63], such as electrophysiological properties, synaptic activity and extensive connective networks, which can be functionally interrogated [21,64].

## Genome-wide CRISPRi screening

**Figure 1.**
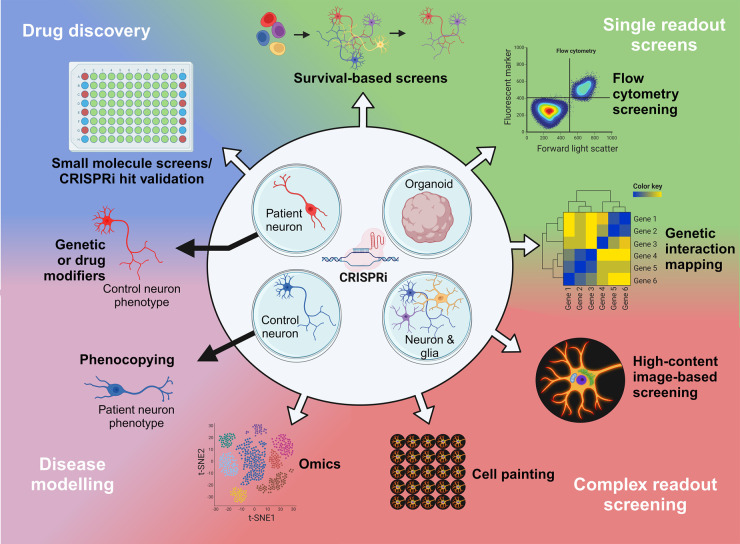
An overview of the utility and applications of CRISPRi in patient and control iPSC-derived cells. CRISPRi can be integrated with various cell models such as control and patient-derived neurons, glia and organoids. This approach can be used to create new disease models from control neurons and understand the effects of disease-causing mutations (or risk modifiers) using patient neurons. In addition, these cell models are applicable to various single-readout screens, such as survival or FACS-based screens, as well as screens with more complex readouts such as genetic interactions, cell painting and -omics. Additionally, CRISPRi can be integrated with drug discovery methods such as small molecule screening and validation of screening hits.

Genome-wide CRISPRn screens offered the first proof that CRISPR technology is amenable to genetic screening and that robust biological information could be obtained at scale [5–8] (Figure 1). By combining specificity, efficacy, scalability and ease of use, both CRISPRn and CRISPRi have enabled a diversity of screen paradigms, which have been comprehensively reviewed elsewhere [65,66].

While CRISPRn was initially found to be more sensitive with identifying hit genes [27], recent improvements have enabled CRISPRi libraries to perform on par with CRISPRn screens [16]. In some cases, the permanent and heterogeneous changes to the genome produced by CRISPRn can be a disadvantage, with studies showing that CRISPRi is less prone to compensation than CRISPRn [31], and that CRISPRi produces a more consistent silencing of genes [30].

Genome-scale screening using CRISPRi technology can be used as a method of pathway or gene discovery, mechanistic interrogation or pharmacological target discovery (Figure 1). Frequently, genome-wide screens identify enriched populations with proliferation or survival advantage [21,22,67,68]. Alternatively, fluorescence-activated cell sorting (FACS) can precede the sequencing readout to separate distinct populations according to a fluorescently-linked phenotype [59,69,70]. This approach is exemplified by a CRISPRi screen targeting both protein-coding genes and long non-coding RNAs to identify regulators of neural induction in iPSCs, as assessed by PAX6 staining [69]. In another example, a recent pooled genome-wide screen used FACS-based sorting to identify genes that modulate α-synuclein expression [71]. The primary CRISPRn screen used a melanoma cell line, and the secondary CRISPRi validation screen used iPSC-derived neurons, demonstrating that combining different cell models and gene perturbation methods can reveal cell-type independent modulators of α-synuclein expression [71].

Additionally, several studies have demonstrated that CRISPRi is highly complementary to the CRISPRa strategy [15,72,73], with use of both technologies in parallel providing deeper insight into biological processes, for example, identifying the lysosomal protein prosaposin as a regulator of lysosomal lipid processing and cellular reactive oxygen species (ROS) [22].

## Integrating CRISPRi with -omics readouts

Whilst the application of CRISPRi to various biological questions has undoubtedly produced a wealth of information about biological pathways and processes (for a screening reference database in the field of neurodegeneration, see CRISPRbrain [22]), another exciting possibility is the combination of CRISPRi screening with single-cell transcriptomics and epigenomics readouts or with high-content imaging readouts, enabling the generation of large, multidimensional datasets (Figure 1).

Approaches relying on single-cell RNAseq (scRNA-seq) such as CROP-Seq [21,74] and Peturb-Seq [75], in which transcriptomic signatures can be used as a powerful multidimensional readout, have been demonstrated in both iPSCs and glutamatergic neurons, highlighting the divergent effects of gene perturbation in iPSCs compared with differentiated neurons [21]. The major advantage of sequencing readouts in pooled CRISPR-based screens is the throughput scalability for genome-wide screening, while still identifying knockdowns enriched within distinct populations. Future application of these CROP-seq-like approaches to single-cell proteomics or lipidomics [76,77] — dependent on methodological improvements in labelling, sensitivity and throughput — are exciting potential avenues of development. Additionally, the integration of multi-omic datasets into a multidimensional functional profile, as previously demonstrated in *Escherichia coli* [78] will offer insight into both the upstream and downstream processes surrounding the perturbation of a disease-associated gene or allow matching of a disease signature to a multi-omic profile.

In addition, CRISPRi may be used as a tool for the validation of differentially regulated genes/proteins or pathways identified by -omics approaches or to recapitulate-omics signatures from patient-derived neuronal models. Current examples of functional validation include CRISPRn, shRNA and small molecule inhibitors of proteins/pathways [43,79,80]; however, the relative benefits of CRISPRi in terms of specificity and scalability may increase the use of this approach.

## High-content imaging readouts

High-content imaging (HCI) has increased in popularity in drug discovery for its ability to generate rich, multiparametric image-based datasets from a singular assay [81]. Longitudinal imaging allows additional temporal resolution of readouts such as neuronal neurite morphology in response to CRISPRi-mediated knockdown of survival genes [21]. Moreover, HCI permits spatial and spectral resolution amenable to multiplexing, demonstrated in an arrayed CRISPRn screen in SH-SY5Y, where endo-lysosomal, tau aggregation and mitochondrial phenotypes were measured in parallel [82]. Alternatively, a novel pooled HCI CRISPRi screening method has been developed, in which cells showing phenotypes such as mitochondrial Parkin recruitment and nuclear TFEB localisation, identified by machine learning, can be selected by FACS with a photoactivatable marker, identifying genes modulating these processes on a genome-wide scale [70].

An extension of HCI is the method of cell painting, which combines the multiplexed labelling of sub-cellular compartments and automated morphological image analysis to generate a multidimensional phenotypic profile described by >1000 features [83]. This morphological fingerprint has the capacity to discriminate between similar but not identical cellular phenotypes that might otherwise be missed in a biased screening format, or cluster drug-induced phenotypes by mechanism of action [84,85]. This approach has already been successful at scale for CRISPRn screening in the U2OS cell line [86].

With the advent of machine learning technology, HCI and cell painting datasets have been used as training datasets in predictive models [70,84,87]. Considering how this could be applied in functional genomics in iPSC-derived neurons opens exciting opportunities for deeper understanding of cellular mechanisms underlying morphological profiles.

## Utilising CRISPRi in iPSC-derived neurons for target validation

In addition to its use as a discovery tool, CRISPRi may be used to validate -omics datasets as well as genetic or small molecule screens (Figure 1). We have recently used CRISPRi as a tool to perform target confirmation arising from a compound screen in SH-SY5Y investigating TFEB and TFE3 translocation [88]. Primary screening revealed PRKD inhibitors as regulating TFEB, however, CRISPRi knockdown of both PRKD2 and/or PRKD3 failed to recapitulate compound-induced phenotypes, or alter the EC_50_ of the small molecule inhibitor [88]. Target validation using CRISPRi, in some cases, may more accurately compare to chemical inhibitors than CRISPRn, as knockdown does not completely eliminate protein levels. This incomplete knockdown may more accurately reflect compounds in development with incomplete inhibition profiles, and can be titrated to confer a range of protein knockdown [30,89]. Alternatively, the level of protein knockdown could be tunable through truncating sgRNA design [90]. An extension of using CRISPRi in compound screening would be a genetic screen for drug sensitivity, quantifying whether genetic knockdown protects from or synergizes drug effect, which can, therefore, reveal further mechanistic insight or uncover putative or novel targets [91].

## Assessing genetic interactions

Genetic interaction (GI) mapping is a powerful technique allowing the systemic and unbiased dissection of complex genetic networks. Initial GI screens were performed using RNAi technology but required the use of complex pooled shRNA libraries to counteract off-target effects and variability in the effectiveness of each shRNA [92–94]. The advantages of CRISPRi, through the ability to produce more homogeneous and specific gene knockdown than shRNA, makes it markedly more accessible to test larger numbers of genes at reduced numbers of sgRNAs per gene. It should be noted that the number of sgRNA permutations necessary for screening large GI screens remains the limiting factor. However, recent advancements, allowing for the random generation of dual-guide vectors simultaneously targeting two genes, are now enabling these CRISPRi GI screens to be performed at genome-wide scale [95].

GI maps have enabled the functional characterisation of genes that were poorly understood and suggested surprising links between seemingly disparate molecular pathways, as well as provided valuable mechanistic insight into complex and adaptive gene networks through the use of single and multiplex readouts [74,96,97]. GI mapping is also making headway in highlighting potential candidate genes for combinatorial therapy in cancer [98–100], where the use of drugs against two targets simultaneously can be useful to overcome resistant cells and reduce individual doses of drugs in an effort to avoid side effects. Similar benefits may be achieved in neurodegeneration by assessing the specific interactions and functional clustering of disease-associated genes.

## Towards arrayed genome-wide CRISPRi

**Figure 2.**
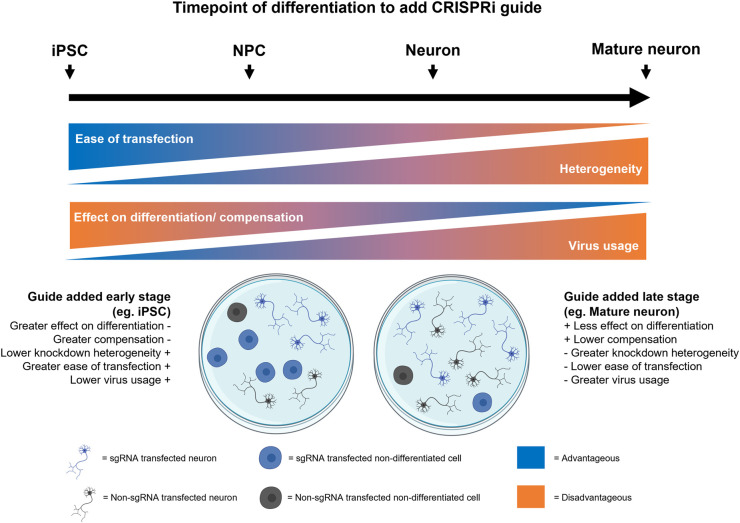
An overview of the technical considerations in the use of CRISPRi in iPSC-derived neuronal models. Several different competing factors need to be considered when determining the optimal time for lentiviral sgRNA delivery during iPSC differentiation to neurons. Cells can be transfected as iPSC, neuronal precursor (NPC), or during neuronal maturation. Early delivery of sgRNAs is more likely to disrupt the differentiation of cells into the final cell type of interest, but there are substantial benefits in terms of transduction efficiency and the ability to create stable gene knockdown lines for downstream disease modelling applications. Late delivery of sgRNAs is complicated by a lower transduction efficiency, but is advantageous in that differentiation efficacy is less affected.

Currently, the advantage of pooled CRISPR screening over arrayed is the capacity to profile the effect of gene perturbations on a genome-wide scale. However, to enable the implementation of assays not compatible with FACS (e.g. HCI) or overcome the bottleneck of cloning/virus production, large-scale arrayed libraries are likely needed. Recently, Yin et al. [101] have demonstrated a high-throughput sgRNA cloning approach (APPEAL; Automated-liquid-Phase-Plasmid-assEmbly-And-cLoning) to generate two arrayed genome-wide libraries with each gene targeted by four sgRNA, each driven off of a different promotor. These libraries enable knockout (CRISPRn) (T.spiezzio) and activation (CRISPRa)/silencing (CRISPRoff) (T.gonfio) and have been validated in both arrayed and pooled screens [101]. However, the logistical challenges and cost of lentivirus production at scale, remain to be resolved to enable widespread adoption, and these challenges are more pertinent for hard-to-infect cell types like iPSC-derived neurons (Figure 2). Pooled genome-wide CRISPRi screening with HCI has been shown to be achievable when combined with downstream selection using photoactivatable markers by FACS (discussed above) [70] offering an alternative solution to this problem, albeit machine learning accuracy is limited to low-frequency phenotypes. Nevertheless, arrayed CRISPR libraries such as T.spiezzio and T.gonfio represent exciting advances that will allow both screening and functional genomics to be carried out at scale, in addition to the creation of isogenic lines that recapitulate disease-associated mutations (discussed below).

## Phenocopying

An important application of CRISPRi is for understanding disease-causing mutations or recapitulating disease phenotypes (phenocopying). CRISPRi is particularly well suited to the study of genes that show differing phenotypes upon partial or complete knockdown of the gene product. CRISPRi, CRISPRn and CRISPRa have all been used to correct/induce phenotypes in patient neurons with multiplications of the *SNCA* gene, comprehensively demonstrating the relationship between the *SNCA* gene and neuronal phenotypes [45,46,102]. The capacity to induce phenotypes in healthy iPSC-derived neurons, has also been demonstrated by CRISPR knock in of familial AD-causing mutations [103]. These studies demonstrate how CRISPRi can be titrated to produce varying levels of silencing [89] — allowing the investigation of dosage effects on disease occurrence. In another example, Hasan et al. [104] have generated isogenic *GRN* KO lines using CRISPRn to recapitulate patient phenotypes in Frontotemporal dementia (FTD) patient-derived neurons carrying *GRN* mutations. These phenotypes include lysosomal alkalinisation and impaired proteostasis in glutamatergic neurons. The ability of CRISPRi to recapitulate these phenotypes will provide mechanistic information on the role of GRN in this process. Sen and Thummer [105] review in further detail the applications of CRISPRn, CRISPRi and CRISPRa in correcting disease-associated phenotypes and phenocopying AD, PD, ALS and HD in iPSC-derived neurons.

A recent effort to create a library of isogenic CRISPR lines (iNDI), recapitulating many of the mutations causing neurodegenerative diseases, provides a benchmark for phenocopying disease mutations using iPSCs [106,107]. This resource will be key for understanding the function and mechanism of many of these genes. However, as discussed previously, CRISPRi libraries or iPSC cells with stable knockdown encompassing these same genes will allow a complementary approach enabling low-effect size mutations to be assessed at pathophysiologically relevant levels. This approach of modelling disease-causing mutations with CRISPRi is potentially enabled through using antibiotic resistance markers, or fluorescent tags [103] to select iPSC-derived cells expressing either the sgRNA and the dCas9 protein, allowing the generation of stable lines.

Using CRISPRi as a genetic screening platform presents the unique opportunity to understand the epistasis of genes which are tagged by single nucleotide polymorphisms (SNPs), where gene expression acts as a modulator for a disease-causing mutation with incomplete penetrance (e.g. *C9orf72*, *LRRK2* or *GBA*). Patient-derived iPSCs or induced models such as stable knockdown/knockout iPSCs from control lines or mouse models [108], can be combined with screening approaches to identify genetic modifiers of disease phenotypes. For example, understanding which genetic modifications result in control iPSC-derived neurons acquiring a ‘patient-cell like’ phenotype would likely be useful in understanding novel genes underlying disease pathways. Likewise, relevant disease mechanisms and therapeutic avenues can be inferred by identifying genetic modifications that rescue a patient-derived iPSC model (Figure 1). Exploring this approach with CRISPRi, in the future, may open the door to understanding modifier genes in monogenic disease, cellular mechanisms underlying polygenic risk-scores and personalised combinatorial therapies.

Importantly, CRISPRi technology coupled with new differentiation protocols for iPSCs has enabled the generation and screening of multiple different cell types, including microglia [109,110] and astrocytes [41]. In particular, by combining these three cell types, it has become possible to create and screen organoid-like assemblies of cells, and at least one group is currently applying this technique to study neuronal survival and the impact of neurodegeneration-associated gene variants in a more complex and physiological model [111].

## Technical challenges and considerations of integrating CRISPRi in iPSC-derived neurons

There are numerous technical factors to consider when integrating CRISPRi with iPSC-derived neurons (Figure 2). In stable dCas9-expressing cells, the timepoint of sgRNA guide delivery is a critical factor and becomes more pertinent if delivering dCas9 by lentivirus alongside sgRNA (Figure 2). At the iPSC stage, cells are easier to transduce leading to more knockdown homogeneity and less virus usage, in addition to the potential to generate stable lines. The transfected cell population can also be purified before differentiation and downstream assays through a co-expressed antibiotic resistance gene or fluorescent marker [21,67]. However, if knockdown is too early, certain perturbations may affect the differentiation or give rise to compensatory mechanisms, impeding the reliability of results, e.g. CRISPRi knockdown of *PPP1R12C* has been demonstrated to provide a proliferation advantage in a pooled CRISPRi survival screen in iPSC-derived neurons [21]. Conversely, delivering the sgRNA later in the differentiation will avoid potential effects on the preceding patterning process and the resulting cell population may have greater knockdown heterogeneity but requires more virus to transduce. Temporal control of knockdown to a specific point in maturation, such as Tet-On inducible expression of dCas9 machinery, as demonstrated in iPSCs and iPSC-derived cardiomyocytes [30] could circumvent early knockdown interfering with the differentiation.

## Conclusions

The advent of CRISPR has enabled unprecedented flexibility to create and edit models of disease. In a similar manner, the ability to generate cell types of interest from iPSCs, which maintain the genetic background of a patient, has had a similarly transformational impact on understanding disease mechanisms. Therefore, the multiple points of convergence of these technologies allow a unique opportunity to address mechanistic and therapeutic challenges for diseases (Figure 1). This is particularly apparent in neurodegenerative diseases in which dysfunction in hard-to-obtain cell types drives disease progression.

Here we highlight that using CRISPRi to manipulate protein levels has emerged as an important tool to understand protein function and the effect of loss-of-function mutations. CRISPRi may be used as a complementary technique to more established tools such as CRISPRn to model decreases in protein expression, whilst offering benefits including decreased off-target effects, decreased compensation and an increased ability to multiplex with selection markers/reporters.

CRISPRi is being used in a greater number of applications, including to understand the role of specific genes and their function, in particular where residual gene product levels or activity produces differing phenotypes to those of a complete loss of expression. This includes many genes identified by genome-wide association studies (GWASs) as contributing to multifactorial diseases such as PD, as well as certain incompletely penetrant disease-causing mutations.

These can be used in both a phenocopying manner to create new disease models using control lines (e.g. *PINK1*/*PRKN*) or to identify modifiers in a patient-derived line bearing mutations with incomplete penetrance (e.g. *C9orf72*/*GBA*/*LRRK2*) or to understand the effects of reducing expression of a disease-associated gene (e.g. *SNCA/GRN*).

The current challenge of CRISPRi is the availability of arrayed genome libraries which provide a barrier to some large-scale screening modalities such as HCI readouts or hit-validation. These have been addressed with established arrayed libraries for CRISPRn and new libraries emerging for CRISPRoff and CRISPRa, but not yet for CRISPRi, for the mammalian genome.

Despite the lack of genome-wide arrayed libraries, targeted libraries covering specific areas of biology, such as genes associated with neurodegenerative diseases, or guides targeting a specific disease-related gene are being used as a complementary tool to dissect gene function. Integration of CRISPRi machinery into the patient and control iPSC lines allows integration of patient models into both gene function and target/drug discovery approaches.

## Perspectives

- Understanding the cellular effects of disease-causing mutations in neurodegeneration is a vital tool for understanding disease mechanisms and therapies. Modulation of gene expression using CRISPR is a key tool to understand the relationship between genetic variants and the functional impact on cells.
- CRISPRi allows robust and sustained knockdown of genes in disease-relevant neuronal and glial cell types and is able identify novel biology using pooled genome-wide screens. CRISPRi may also be used as a tool to knock down disease-causing mutations in relevant cell types to phenocopy patient neurons.
- Multiplexing CRISPR guides with imaging/selection markers will enable innovative applications of CRISPRi. These applications include GI mapping and -omic readouts at scale in addition to specific applications such as cell painting. As well as identifying genes which modify the risk conferred by mutations with incomplete penetrance.

## Open Access

Open access for this article was enabled by the participation of the University of Oxford in an all-inclusive *Read & Publish* agreement with Portland Press and the Biochemical Society under a transformative agreement with JISC.

## Abbreviations

AD: Alzheimer's disease
ALS: amyotrophic lateral sclerosis
CRISPRa: CRISPR activation
CRISPRi: CRISPR interference
CRISPR: clustered regularly interspersed short palindromic repeat
CRISPRn: CRISPR/Cas9 system
dCas9: nuclease-dead Cas9
FACS: fluorescence-activated cell sorting
HCI: high-content imaging
HD: Huntington's disease
iPSCs: induced pluripotent stem cells
PD: Parkinson's disease
RNAi: RNA interference
sgRNAs: single guide RNAs
RNP: ribonucleoprotein
shRNA: small hairpin RNA
TSS: transcription start site
